# T-type Ca^2+^ channels regulate the exit of cardiac myocytes from the cell cycle after birth

**DOI:** 10.1016/j.yjmcc.2013.05.016

**Published:** 2013-06-04

**Authors:** Fang Wang, Hui Gao, Hajime Kubo, Xiaoxuan Fan, Hongyu Zhang, Remus Berretta, Xiongwen Chen, Thomas Sharp, Timothy Starosta, Catherine Makarewich, Ying Li, Jeffrey D. Molkentin, Steven R. Houser

**Affiliations:** a Cardiovascular Research Center, Temple University School of Medicine, 3500 North Broad Street, Philadelphia, PA 19140, USA; b Department of Pediatrics, University of Cincinnati, Cincinnati Children's Hospital Medical Center, Cincinnati, OH 45229, USA

**Keywords:** T-type Ca^2+^ channels, Cardiac myocytes, Cell cycle

## Abstract

T-type Ca^2+^ channels (TTCCs) are expressed in the fetal heart and then disappear from ventricular myocytes after birth. The hypothesis examined in this study was the α1G TTCCs' influence in myocyte maturation and their rapid withdrawal from the cell cycle after birth.

**Methods:**

Cardiac myocytes were isolated from neonatal and adult wild type (WT), α1G−/− and α1G over expressing (α1GDT) mice. Bromodeoxyuridine (BrdU) uptake, myocyte nucleation, cell cycle analysis, and T-type Ca^2+^ currents were measured.

**Results:**

All myocytes were mono-nucleated at birth and 35% of WT myocytes expressed functional TTCCs. Very few neonatal myocytes had functional TTCCs in α1G−/− hearts. By the end of the first week after birth no WT or α1G−/− had functional TTCCs. During the first week after birth about 25% of WT myocytes were BrdU+ and became bi-nucleated. Significantly fewer α1G−/− myocytes became bi-nucleated and fewer of these myocytes were BrdU+. Neonatal α1G−/− myocytes were also smaller than WT. Adult WT and α1G−/− hearts were similar in size, but α1G−/− myocytes were smaller and a greater % were mono-nucleated. α1G over expressing hearts were smaller than WT but their myocytes were larger.

**Conclusions:**

The studies performed show that loss of functional TTCCs is associated with bi-nucleation and myocyte withdrawal from the cell cycle. Loss of α1G TTCCs slowed the transition from mono- to bi-nucleation and resulted in an adult heart with a greater number of small cardiac myocytes. These results suggest that TTCCs are involved in the regulation of myocyte size and the exit of myocytes from the cell cycle during the first week after birth.

## 1. Introduction

T-type Ca^2+^ channels (TTCCs) are expressed in the fetal heart during development [1–3], but their expression deceases after birth [4–6]. These channels are not present in the ventricle of most normal adult animals but are re-expressed in cardiac disease states and are associated with cardiac hypertrophy [7]. While the biophysical properties of TTCCs are well described [2,8], their biological functions are not well known. In the heart there is evidence that TTCCs are involved in the generation of pacemaker potentials [9], but do not appear to play any substantial role in excitation–contraction coupling [2,8]. In smooth muscle and in cancers there is a linkage between the presence of TTCCs and cellular proliferation [10–12], suggesting that Ca^2+^ influx through TTCCs influences the cell cycle.

Cardiac myocytes are proliferative in the fetal heart but soon after birth ventricular myocytes become terminally differentiated and bi-nucleated and lose their ability to reenter the cell cycle [13–15]. There is an association between fetal myocyte proliferation and the presence of TTCCs and the reduced expression of TTCCs after birth is associated with the exit of ventricular myocytes from the cell cycle.

The working hypothesis of this study is that α1G TTCCs are involved in the regulation of myocyte size and cell cycle after birth. The objectives of the present study were (1) to define the relationship between the reduction of TTCC expression in normal cardiac myocytes after birth and their exit from the cell cycle in the first week after birth, and (2) to determine the effects of loss of α1G TTCCs (α1G−/−) on myocyte size, proliferation, binucleation and the exit of myocytes from the cell cycle after birth.

The experiments performed in the present study showed that TTCC currents are observed in about 35% of ventricular myocytes immediately after birth in normal hearts but almost no myocytes had functional TTCCs by the end of the first week of life. The loss of α1G TTCCs in the normal, wild type mouse heart was associated with an increase in the percentage of binucleated myocytes and an increase in myocyte size. In α1G−/− mice there were very few (<5%) myocytes with any TTCC current at birth and no myocytes with TTCCs were found in seven day old animals. There was also a slower development of myocyte binucleation after birth and myocyte size was smaller in α1G−/− neonatal mice. In the adult α1G−/− mouse, heart size was normal, but myocyte size was smaller than in normal animals and more adult α1G−/− myocytes were mononucleated. These finding suggest that the loss of α1G TTCCs slowed the exit of adult cardiac myocytes from the cell cycle, leading to an adult heart with an increased number of smaller, mononucleated cardiac myocytes. Collectively these studies suggest that Ca^2+^ influx through α1G TTCCs regulates the exit of cardiac myocytes from the cell cycle and their growth during the first week after birth.

## 2. Material and methods

### 2.1. Mice

Wild type C57BL/6 mice were obtained from Jackson Laboratories. α1G gene-targeted knockout mice (α1G−/−) were provided by Dr. Jeffrey Molkentin [16]. Cardiac-specific α1G subunit over-expressing transgenic mice (α1G double transgenic, DT) were generated by using a modified murine α-myosin heavy chain (α-MHC) promoter expression vector as described previously [17,18]. In the absence of tetracycline/doxycycline (Dox), responder transgene α1G TTCC expression is permitted in the presence of tetracycline transactivator (tTA) protein. Heterozygous tTa and α1G breeding pairs produced α1GDT mice that were deprived of Dox food after birth to activate gene expression [19,20]. The experiments followed guidelines provided by the National Institutes of Health Guide for the Care and Use of Laboratory Animals. All procedures were approved by the Institutional Animal Care and Use of Committee at Temple University School of Medicine.

### 2.2. Neonatal mouse cardiomyocyte isolation and culturing

Neonatal cardiomyocytes were prepared from pups on days 1 or 7 after birth. Neonatal mice were decapitated, the chest cavities were cut open and the mouse hearts were removed. Subsequently, cardiomyocytes were dispersed by incubation with DNase (0.2 mg/ml) and trypsin (3 mg/ml) buffer solution containing the following in mM: NaCl 137, KCl 5.36, MgSO_4_-7H_2_O 0.81, dextrose 5.55, KH_2_PO_4_ 0.44, Na_2_HPO_4_-7H_2_O 0.34, and HEPES 20, pH 7.5, and the cellular suspension was filtered through a polypropylene macroporous filter (mesh opening: 100 μm). The suspension was then centrifuged at 1000 rpm for 5 min, the cellular pellet was suspended in MEM with Hank's balanced solution (Gibco, Cat# 11575-032) with 5% FBS and penicillin–streptomycin (50 μg/ml). Cellular suspensions were used in flow cytometry experiments. For cell cycle studies the cell suspension was preplated at 37 °C for 1 h in 1% CO_2_ to reduce non-myocyte contamination. 2 × 10^5^myocytes were then plated in polystyrene, nonpyrogenic 12-well culture plates which had been coated with 1% gelatin at room temperature for 2 h. Neonatal myocytes were incubated in 1% CO_2_ at 37 °C for 24 h before immuno-fluorescence or electrophysiology studies.

### 2.3. Adult cardiomyocyte Isolation and culture

Mice were anesthetized with sodium pentobarbital (0.5 mg/100 g, i.p.). Hearts were perfused via the aorta with Tyrode solution [composition in mM: glucose 10, HEPES 5, CaCl_2_ 0.02, KCl 5.4, NaCl 150, MgCl_2_ 1.2, and sodium pyruvate 2, pH 7.4] containing collagenase (300 U/ml). After 6–7 min of perfusion the heart softened and the ventricles were minced and filtered through macroporous filter (mesh opening: 100 μm). Isolated ventricular myocytes were equilibrated in Tyrode solution [composition in mM: glucose 10, HEPES 5, CaCl_2_ 1.0, KCl 5.4, NaCl 150, MgCl_2_ 1.2, and sodium pyruvate 2, pH 7.4] and 0.5% bovine serum albumin. Myocytes were seeded (4 × 10^4^) in polystyrene, nonpyrogenic 12-well culture plates precoated with 40 μg/ml laminin.

### 2.4. Immunohistochemistry

Newly formed DNA was identified by BrdU (Roche) incorporation into the DNA of cardiomyocytes which were isolated from both wild type (WT) and α1G−/− mice. For BrdU incorporation experiments, neonatal mice were injected with 1 mg BrdU per 6 g body weight (IP). BrdU injection was performed twice a day for 7 days. On day 7, neonatal mice were decapitated, the chest cavities were cut open and the mouse hearts were removed. Hearts were fixed in formalin. Using standard procedures, tissue sections (5 μm) were incubated with BrdU antibodies (target for cell proliferation), cardiac α-actin antibodies and DAPI (to identify nuclei). BrdU+ myocyte nuclei were identified as we have described previously [21].

### 2.5. Myocyte cross-sectional area

Hearts were perfusion fixed with 10% formalin [2], embedded in paraffin and 5 μm thick sections were cut and stained with hematoxylin/eosin [22]. Cardiomyocyte cross-sectional area was measured from areas that were clearly cut in cross section. Images were analyzed using NIH Image J software system.

Isolated adult ventricular cardiomyocytes were cultured on cover slips at room temperature for 1.5 h, then fixed in 4% paraformaldehyde solution at room temperature for 10 min and permeabilized in 0.25% Triton X-100 immediately before labeling with antibodies. Staining of cardiac α-actinin and DAPI was performed to detect myocytes and nuclei. Cardiomyocytes were cultured and fixed on 4 cover slips mounted onto slides and 12 random fields from each cover slip were observed with a confocal microscope (Nikon). Images were analyzed with EZ-C1 FreeViewer (Nikon) and ImageJ (NIH) software. Comparison was made between the two groups WT (n = 3; n: number of mice) and α1G−/− (n = 3).

Isolated neonatal ventricular cardiomyocytes were cultured at 37 °C and 1% CO_2_ for 48 h, then fixed in 4% paraformaldehyde solution and permeabilized in 0.05% Triton X-100 immediately before labeling with antibodies directed against α-actinin. Staining of α-actinin and DAPI was performed to detect myocytes and their nuclei respectively. Cardiomyocytes were cultured and fixed on 4 cover slips, cover slips were mounted onto slides and 10 random fields for each cover slip were observed. Images were analyzed with EZ-C1 FreeViewer (Nikon) and ImageJ (NIH) software. Comparison was made between the two groups WT (n = 10 on day 1, n = 10 on day 7; n: number of neonatal mice) and α1G−/− (n = 8 on day 1, n = 9 on day 7).

### 2.6. Flow cytometry

Flow cytometry was performed on myocytes from neonatal mouse hearts: neonatal mouse hearts were isolated under a dissecting microscope, gently minced and then dissociated to single cell solution with DNase (0.2 mg/ml) and trypsin (3 mg/ml) buffer solution [composition in mM: NaCl 137, KCl 5.36, MgSO_4_-7H_2_O 0.81, dextrose 5.55, KH_2_PO_4_ 0.44, Na_2_HPO_4_-7H_2_O 0.34, and HEPES 20, pH 7.5]. Cells were fixed with 70% ethanol, pH 2, permeabilized with 0.05% Triton X-100 and incubated with a mouse anti-actin antibody. Cardiomyocytes were identified by primary antibody (anti-actin) and secondary antibody (APC). Age matched WT and α1G−/− mice were studied on Day 1 (neonatal mouse on day 1) (n = 12 for WT, n = 20 for α1G−/−, n: number of the neonatal mouse hearts) or on Day 7 after birth (n = 28 for WT, n = 20 for α1G−/−).

### 2.7. BrdU and cell cycle (DNA content) analysis of neonatal mouse cardiomyocytes

Neonatal mice were given an intraperitoneal BrdU injection as described previously. BrdU incorporation was measured using instructions within a BrdU intracellular staining kit (BD Pharmingen). Cell cycle (DNA content) analysis was performed by staining DNA with 7-amino actinomycin (7-AAD) (BD Pharmingen) using standard approaches. Flow cytometry data were analyzed using Diva and FlowJo software.

### 2.8. Electrophysiology

L- (I_CaL_) and T-type (I_CaT_) Ca^2+^ currents were measured in a K-free and Na-free solution [2]. Neonatal cardiomyocytes attached to cover slips were placed in a chamber mounted on an inverted microscope (Nikon Diaphot) and perfused with 1 mM Ca^2+^ containing Tyrode solution. The temperature of inflow and outflow solutions was maintained at 36 ± 1 °C. Pipettes were filled witha Cs-containing solution [N-methyl-d-glucamine (NMDG) 10 mM, Cs-aspartate 130 mM, HEPES 10 mM, MgCl_2_ 1 mM, tetraethylammonium chloride 20 mM, Tris-ATP 2.5 mM and EGTA 10 mM, pH 7.2] [2]. Once gigaseals were obtained the patch was ruptured and the cell was dialyzed for 30 s. The extracellular bath was then changed to one containing: CaCl_2_ 2 mM, CsCl 5.4 mM, 4-aminopyridine 2 mM, MgCl_2_ 1.2 mM, glucose 10 mM, HEPES 5 mM, NMDG 150 mM and pH 7.4 with CsOH. An Axopatch 2B voltage-clamp amplifier was used to control the membrane potential and membrane currents were analyzed with pClamp 8 software. Total cell membrane T-type currents and L-type currents were measured from a holding potential of −90 mV using square wave pulses from −70 mV to +50 mV, in 10 mV increments. I_CaL_ was measured from a holding potential of −50 mV using identical square wave pulses. I_CaT_ was calculated by subtracting the I_CaL_ measured from a holding potential of −50 mV from the total Ca^2+^ current measured from a holing potential of −90 mV.

### 2.9. Statistics

Data were expressed as mean ± SEM. Differences between WT, α1G−/− and α1GDT were compared using unpaired Student's t-test. For multiple repeat comparisons between WT, α1G−/− and α1GDT, an ANOVA was performed. A p value of ≤0.05 was considered significant.

## 3. Results

### 3.1. T-type Ca^2+^ currents during the first week of life and in adult mice

**Fig. 1:**
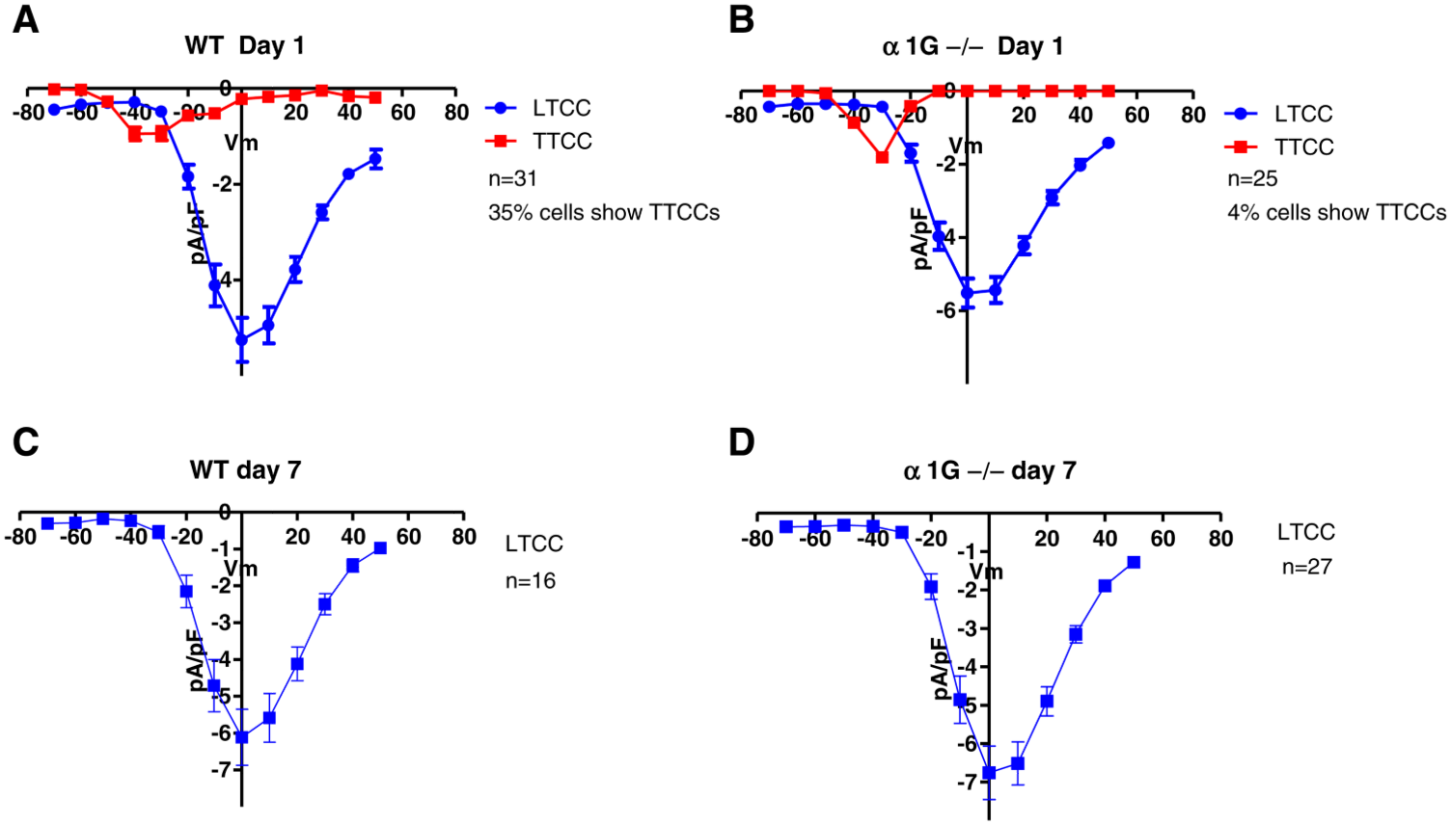
T- (I_CaT_) and L- (I_CaL_) type Ca^2+^ currents in neonatal mouse ventricular myocytes (NMVMs) 1 and 7 days after birth. (A) On day 1, I_CaT_ was detected in 35% of WT myocytes, and (B) in only 4% of α1G−/− NMVMs. On day 7, no I_CaT_ was detected in WT (C) or (D) α1G−/− myocytes. I_CaL_ was not different in either group at either 1 or 7 days after birth (N: # of cells).

TTCC and LTCC currents were measured in neonatal myocytes 1 and 7 days after birth in WT and α1G−/− mice (Fig. 1). All myocytes exhibited robust I_CaL_ and there were no differences in I_CaL_ density between WT and α1G−/− myocytes at either day 1 or day 7 after birth (Fig. 1). I_CaT_ was only found in 35% of WT neonatal myocytes on day 1 and we did not find any WT myocytes with I_CaT_ at day 7 (Figs. 1A, C). Significantly fewer myocytes exhibited a measureable I_CaT_ in α1G−/− myocytes. On day 1 only 1 myocyte (4% of those studies) had a measureable I_CaT_. No I_CaT_ was found at day 7 in the 27 α1G−/− myocytes studied. These results show that there is a significant loss of Ca^2+^ influx through TTCC in WT myocytes and α1G−/− myocytes have little or no Ca^2+^ entry via TTCCs during the first week of life. The I_CaT_ found in the single α1G−/− myocyte at day 1 likely represents current through α1H TTCC, the other channel that has been found in neonatal hearts [23,24]. These results also suggest that the α1G TTCC is the major TTCC expressed in the neonatal mouse heart during the first week after birth and show that loss of α1G does not induce a compensatory increase in the expression of other TTCCs.

### 3.2. BrdU incorporation into WT and α1G−/− neonatal myocytes

**Fig. 2:**
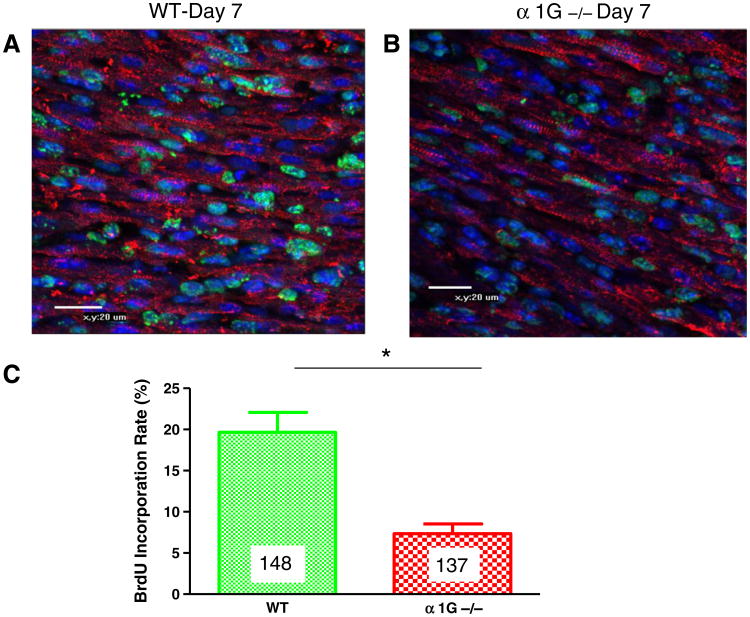
BrdU+ myocytes during the first week of life: Representative confocal images (A, B) of 7 day old WT (n = 148, N = 3) and α1G−/− (n = 137, N = 3) hearts. BrdU is in green, DAPI is in blue and cardiac α-actin is in red. Merged images are shown. Average data is shown in C. There were significantly fewer BrdU+ myocyte nuclei in α1G−/− hearts. *p < 0.005, n: # of myocytes, N: # of hearts.

**Fig. 3:**
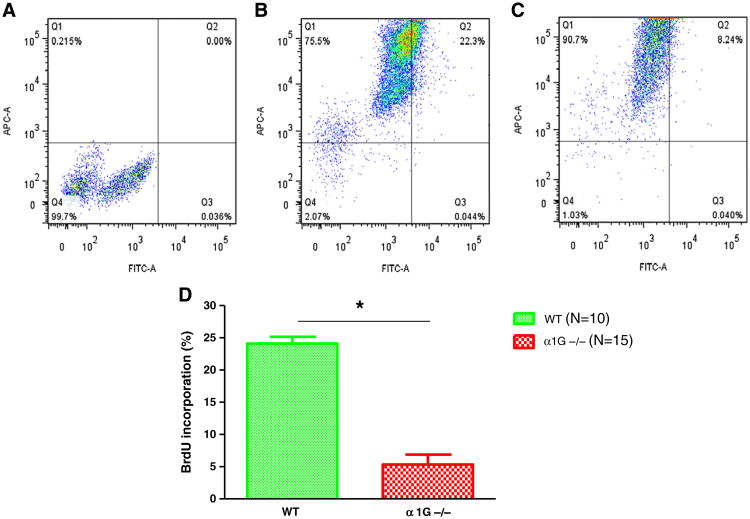
BrdU incorporation into DNA of NMVMs during the first week after birth determined with flow cytometry. NMVMs were isolated from BrdU injected mouse hearts 7 days after birth. NRVMs were stained for cardiac actin-APC and BrdU-FITC to identify myocytes within newly formed DNA. (A) Unstained control NRVMs were used to set thresholds for identifying NRVMs with BrdU+ DNA. This approach was confirmed versus studies with animals that had not been BrdU injected, but myocytes were stained for BrdU (see Supplemental Fig. 1). (B) In wild type NMVMs 22.3% were BrdU+. (C) In α1G−/− NMVMs 8.24% were BrdU+. (D) Average data from 10 WT and 15 α1G−/− NRVM preparations. There were significantly more BrdU+ myocytes in WT versus α1G−/− myocytes. *p < 0.05 (N: # of hearts).

Neonatal mice were injected with BrdU for 7 days and then BrdU+ myocyte nuclei were measured in the intact heart, with immunostaining (Fig. 2), or in isolated myocytes with flow cytometry (Fig. 3). Immunostaining of the intact heart showed a large number of BrdU+ myocytes and non-myocytes. In WT 7 day old hearts, 20% of the myocyte nuclei were BrdU+ with the BrdU labeling technique employed (Fig. 2). There were significantly fewer BrdU+ myocyte nuclei (7%) in 7 day old α1G−/− hearts (Fig. 2).

Defining myocyte specific nuclei in tissue sections can be challenging [21], so to confirm the results of studies with tissue sections we measured the % of BrdU+ myocyte nuclei in the experiments with isolated myocytes using flow cytometry (Fig. 3). These studies also showed that there were significantly more BrdU+ myocytes in WT hearts (24%) than in α1G−/− (4%) hearts (Fig. 3). These two independent techniques yielded very similar results and support the conclusion that the % of 7 day old myocytes with BrdU+ nuclei was significantly smaller in α1G−/− hearts.

### 3.3. Cell cycle (DNA content) analysis with flow cytometry

**Fig. 4:**
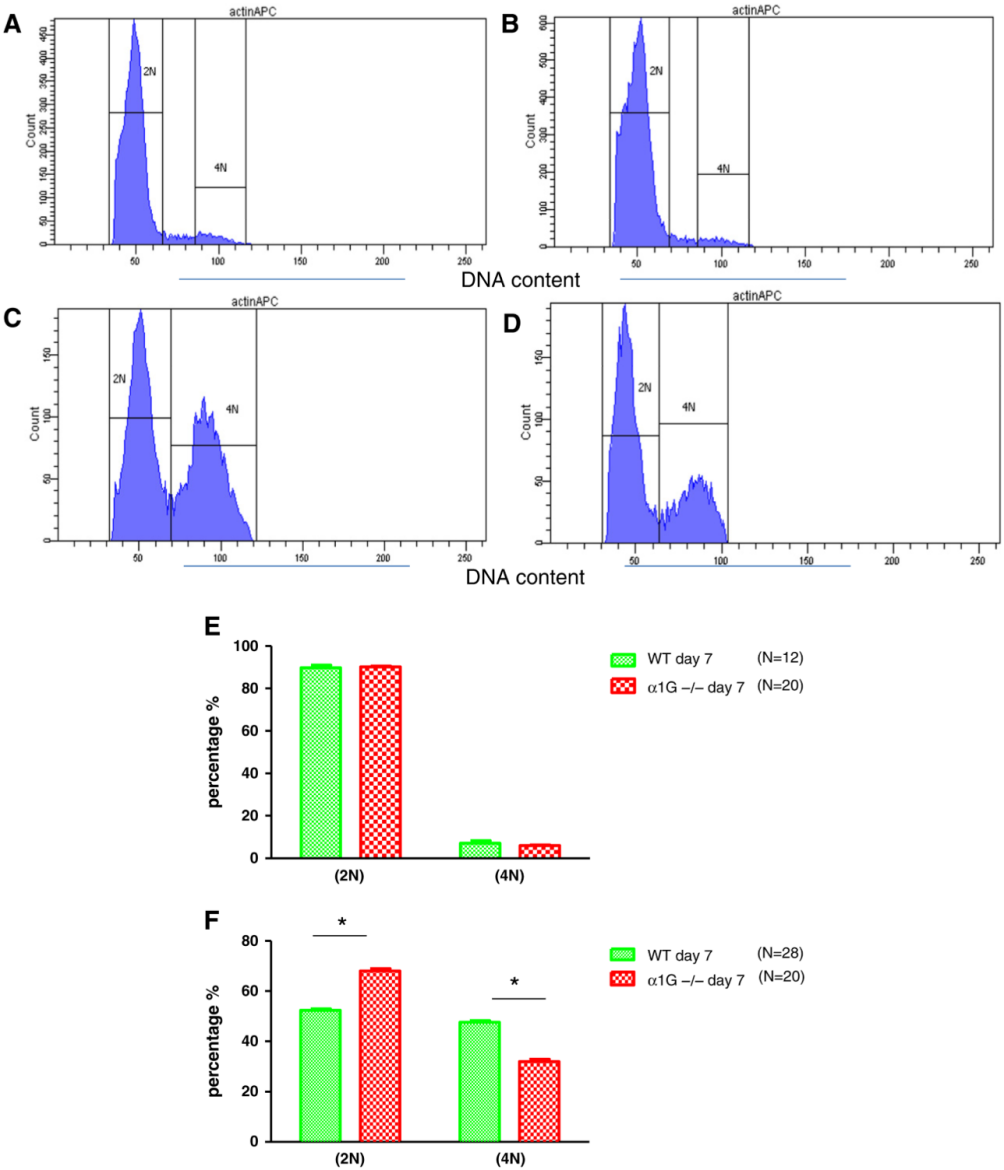
Representative DNA quantity analysis of isolated NMVMs using flow cytometry. NMVMs were isolated on day 1 and day 7 after birth and then DNA content analysis was performed. Single cells were gated. An antibody against cardiac actin was used to identify NMVMs. (A) wild type NMVMs on day 1. (B) α1G−/− NMVMs on day 1. (C) Wild type NMVMs on day 7. (D) α1G−/− NMVMs on day 7. Increased DNA content was observed in NMVMs at 7 days after birth versus 1 day after birth (within groups). There were more WT than α1G−/− myocytes with 4N DNA content. (E) There was no significant difference in DNA content between wild type and α1G−/− on day 1 after birth. (F) The percentage of NMVMs with 2N DNA content decreased in both WT and α1G−/−, however there were significantly fewer α1G−/− NMVMs with 4N DNA content on day 7 after birth. *p < 0.001 (N: # of hearts).

**Fig. 5:**
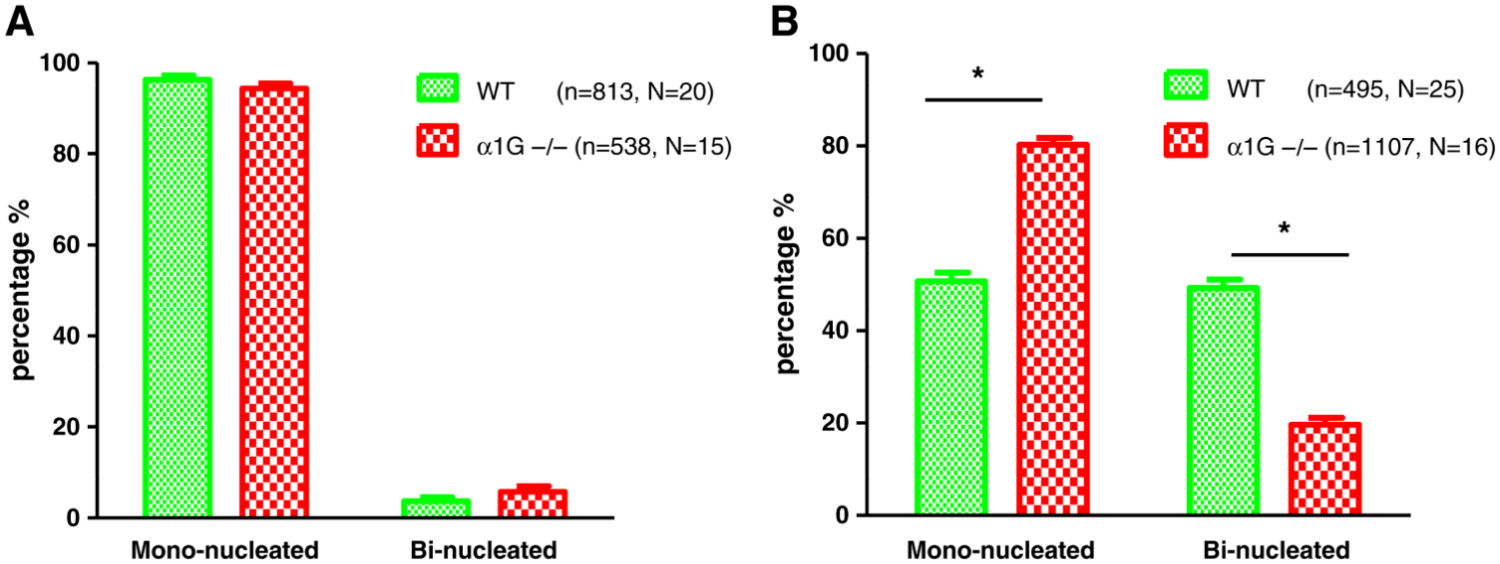
Nucleation of NMVMs isolated on day 1 and day 7 after birth: (A) Almost all myocytes were mono-nucleated on Day 1 after birth in both α1G−/− and WT mice. (B) By day 7 after birth, a significantly greater % WT NMVMs had become bi-nucleated than in α1G−/− myocytes. *p < 0.005, n: # of cells, N: # of hearts.

**Fig. 6:**
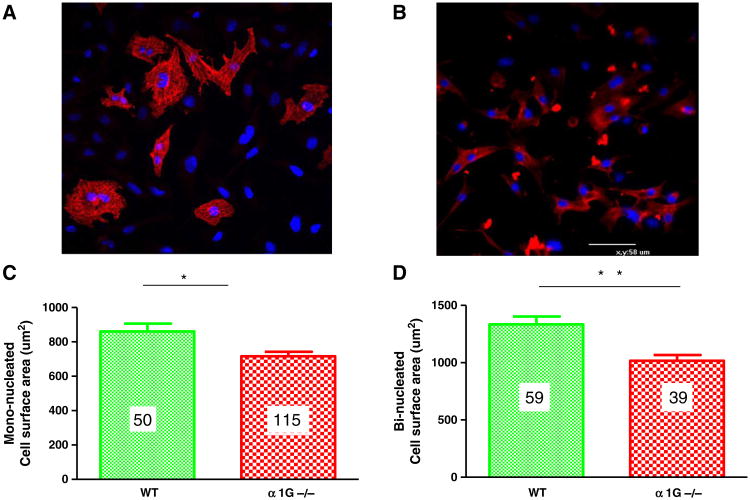
Cell surface area in wild type (n = 109, N = 25) and α1G−/− (n = 154, N = 16) NMVMs on day7 after birth. Representative images of wild type (A) and (B) α1G−/− NMVMs on day 7. Cardiac actin is in red and DAPI is in blue. α1G−/− NMVMs were significantly smaller than WT. (C) Cell surface area of WT mono-nucleated NMVMs (n = 50) was larger than in α1G−/− (n = 115). (D) Cell surface area of WT bi-nucleated NMVMs was greater (n = 59) than in α1G−/− (n = 39). *p < 0.005; **p < 0.001, n: # of NMVMs, N: # of hearts.

BrdU incorporation into myocyte DNA during the first week of life could represent myocyte proliferation or the doubling in myocyte DNA content that takes place with binucleation, which has been associated with exit from the cell cycle [25,26]. To examine these issues we first performed cell cycle (DNA content) analysis of neonatal myocytes from WT and α1G−/− neonatal mouse hearts at days 1 and 7 after birth (Fig. 4). On Day 1 after birth more than 90% of all myocytes from WT and α1G−/− hearts were in the G1/G0 phase of the cell cycle with a 2N compliment of DNA. These results are consistent with the idea that most neonatal myocytes were mononucleated and newly formed, with some possibility that they are still capable of cell cycle activity. Cell cycle (DNA content) analysis showed that WT and α1G−/− myocytes made a 2N to 4N transition (Fig. 4) by Day 7. However, significantly fewer WT (52%) than α1G−/− (68%) myocytes were still in the G1/G0 (2N) phase of the cell cycle on day 7 after birth, with more WT myocytes in the G2 (4N) phase. These results show a 2N to 4N transition for cardiac myocytes in the first week after birth with a significantly slower transition in α1G−/−myocytes (Fig. 4). These changes in DNA content were closely associated with differences in nucleation (Fig. 5) and cell size (Fig. 6) between WT and α1G−/− myocytes during the first week after birth (see below).

### 3.4. Myocyte nucleation changes during the first week of life

Myocytes were isolated from WT and α1G−/− hearts 1 and 7 days after birth to unambiguously determine the percentage of mono and binucleated myocytes (Fig. 5). These studies provide a third independent test of the changes in myocyte nucleation and DNA content after birth. These experiments showed that the majority (>95%) of WT and α1G−/− myocytes were mononucleated on Day 1 after birth (Fig. 5). By Day 7 the % of mononucleated myocytes had fallen to 50% in the WT but only to 80% in α1G myocytes. Changes in nucleation between and within both groups were significant when Day 1 versus Day 7 data were compared. In addition there was a significantly smaller reduction in the % of mononucleated myocytes in α1G−/− versus WT myocytes during the first week after birth. The results of these three independent sets of experiments all support the idea that in the absence of α1G TTCCs neonatal myocytes has a slower 2N to 4N transition (mono- to bi-nucleation).

We also determined if there were differences in myocyte size by the end the first week of life (Fig. 6) in WT and α1G−/− myocytes. The size of mononucleated and binucleated myocytes of 7 day old WT myocytes was significantly larger than in α1G−/− myocytes. Collectively these data show that in the first week after birth, neonatal α1G−/− myocytes have less BrdU incorporation, make a slower 2N to 4N transition, make a slower mono- to binucleated transition and are smaller than their WT counterparts. These results support the idea that α1G TTCCs regulate cell cycle activity, cell growth, and withdrawal from the cell cycle in neonatal myocytes.

### 3.5. T-type Ca^2+^ current, cell size and nucleation in the adult WT, α1G and α1GDT hearts

We next explored if any of the differences in nucleation and myocyte size observed in neonatal myocytes were still present in the adult animal. These studies were performed at 2 months of age at a time when we have shown that these hearts have normal (WT and α1G−/−) or enhanced (α1GDT) [2] function and normal tissue structure with no fibrosis [27]. We added the gain of function animals (α1GDT) to the adult animal analyses to enhance the scope of the work. These animals were not used in neonatal studies because α1GDT expression was not activated until after birth. In addition, there is modest α-MHC-mediated expression in the neonatal time frame and our breeding strategy yielded only 25% DT animals.

Ca^2+^ currents were measured in adult WT and α1GDT myocytes (Supplemental Fig. 2). I_CaT_ was not found in any WT (or in α1G−/−, not shown) adult myocytes. Large I_CaT_ was observed in α1GDT myocytes, as we have reported previously [2].

**Fig. 7:**
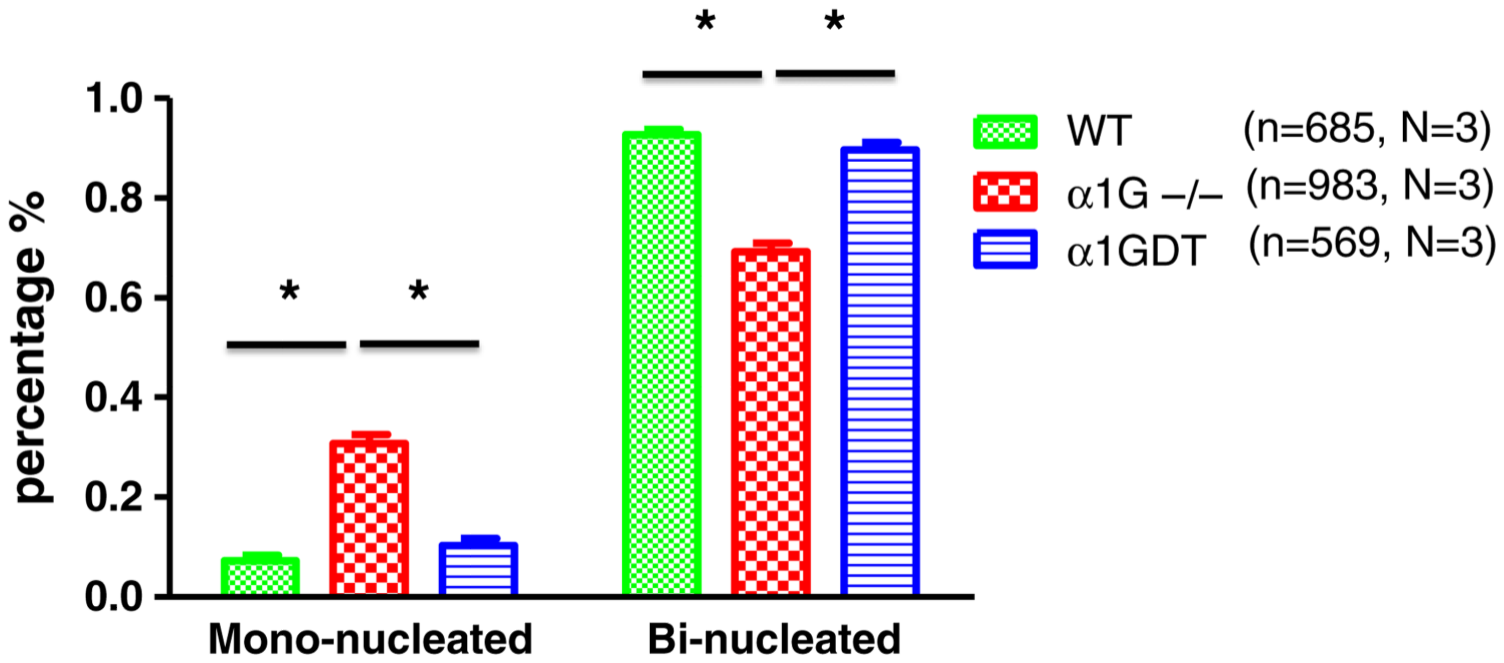
Nucleation analysis of 2 months old WT, α1G−/− and α1GDT myocytes. The percentage of bi-nucleated myocytes was >90% in WT and α1GDT myocytes. The % of binucleated α1G−/− myocytes was significantly less than in WT and α1GDT. *p < 0.001 (n: # of cells, N: # of hearts).

**Fig. 8:**
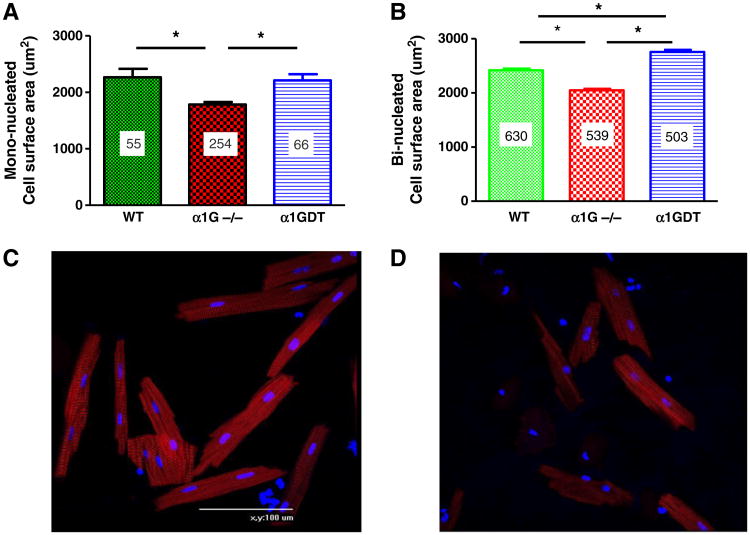
Cell surface area was measured in myocytes isolated from wild type (N = 3), α1GDT (N = 3) and α1G−/− (N = 3) 2 months old mice. (A) Cell surface area of mono-nucleated myocytes in wild type (n = 55) and in α1GDT (n = 66) was larger than in α1G−/− (n = 254). (B) Cell surface area of bi-nucleated myocytes from wild type (n = 630) and α1GDT (n = 503) hearts were larger than in α1G−/− (n = 539). (C) Representative wild type and (D) α1G−/− myocytes from 2 month old hearts are shown. Cardiac α-actin is in red and DAPI is in blue. *p < 0.0001, n: # of myocytes, N: # of hearts.

We have previously reported that heart weight, normalized to body weight or tibial length, is not different in 2 month old α1G−/−, α1GDT and WT mice [27]. In the present study we isolated myocytes from these hearts to measure myocyte size (surface area) and myocyte nucleation (Figs. 7, 8). Measurements were made in mono- and bi-nucleated myocytes from all three groups of mice. Both mono- and bi-nucleated myocytes from α1G−/− hearts were significantly smaller than those from WT and α1GDT hearts. Bi-nucleated α1GDT myocytes were significantly larger than those from WT and α1G−/− hearts (Fig. 7).

Myocyte nucleation (mono- versus bi-nucleation) was also measured in myocytes isolated from WT, α1G−/− and α1GDT animals. More than 90% of all myocytes were binucleated in WT and α1GDT hearts. The % of mononucleated myocytes was significantly greater in α1G−/− myocytes versus that of the other two groups (Fig. 8), consistent with the results in the neonatal time period. Collectively these results suggest that myocyte size and number are influenced by α1G TTCCs.

In summary, these experiments show that neonatal myocytes without α1G TTCC are smaller than normal and appear to have a slower than a normal exit from the cell cycle. The adult heart has a normal size but has myocytes that are smaller than normal and there is a greater percentage of mononucleated myocytes.

## 4. Discussion

T-type Ca channels are expressed in cardiac myocytes in the fetal and neonatal hearts [1], but are not normally found in adult ventricular myocytes [8]. These channels are re-expressed in a subpopulation of adult myocyte subjected to pathological stress [7,28]. The functional role of TTCCs in ventricular myocytes is not well known. While TTCCs allow voltage dependent Ca^2+^ current entry they are not involved in the excitation–contraction coupling [2,29]. There is some data in non-cardiac cell types showing that Ca^2+^ influx through TTCCs is linked to cell proliferation and to specific phases of the cell cycle [30,31,12,32]. In the present experiments we explored the association between TTCC expression in the neonatal heart during the first week after birth, myocyte growth in neonatal cardiac myocytes and the exit of myocytes from the cell cycle.

The major findings of this study are that 1) on Day 1 after birth about 35% of ventricular myocytes had functional TTCCs, and this % decreases during the first week of life. 2a) by two months of age normal ventricular myocytes do not have functional TTCCs; 2b) α1G TTCCs appear to be the major TTCC expressed during the first week of life. 3) BrdU is incorporated into ventricular myocytes during the first week of life and this is associated with a 2N to 4N transition and binucleation, representing myocyte withdrawal from the cell cycle. 4) Loss of α1G TTCCs was associated with a slower transition from mononucleation to binucleation and reduced BrdU incorporation in the first week after birth; and 5) loss of α1G TTCC resulted in an adult heart with more myocytes than are found in the WT heart and these myocytes are smaller and a greater % are mononucleated. Collectively these data suggest that Ca^2+^ influx through α1G TTCCs regulates the transition from a proliferative fetal myocyte to a terminally differentiated binucleated adult ventricular myocyte during the first week of neonatal life.

### 4.1. TTCCs and BrdU incorporation in myocytes in the first week after birth

Our studies show that 1 day after birth most of the myocytes in the neonatal heart are mononucleated and a fraction (about 35%) have functional TTCCs and almost all myocytes are mononucleated. By Day 7 after birth very few myocytes express TTCCs and many of the myocytes have become binucleated, which we interpret as a sign that they have withdrawn from the cell cycle [26,33]. When BrdU was infused during the first week of life about 25% of the myocytes were labeled. Our interpretation of all of these data is that the primary form of DNA synthesis during the first week of life is associated with a 2N to 4N transition (which we document) and this is associated with a loss of TTCC activity. Support for a cause and effect relationship between TTCCs and these changes in myocyte nucleation was gained from experiments with α1G−/− animals. These experiments showed that like WT myocytes, most α1G−/− myocytes were mononucleated on Day 1 after birth, but a very small fraction of myocytes expressed TTCC currents. Presumably these myocytes expressed the α1H TTCC [1,34]. On Day 7 after birth we failed to find any α1G−/− myocytes with TTCC currents. We showed that during the first week of neonatal life, fewer α1G−/− myocytes made the 2N to 4N transition, less were binucleated and a smaller % were labeled with BrdU. Myocytes from these α1G−/− hearts were smaller than WT myocytes at 7 days after birth. These results are consistent with the idea that Ca^2+^ influx through TTCCs regulates myocyte growth, the 2N to 4N (binucleation) transition and the associated exit of cardiac myocytes from the cell cycle. If true this would suggest that α1G−/− hearts have a longer than a normal time period during which their ventricular myocytes can proliferate.

### 4.2. TTCCs, myocyte number and myocyte nucleation in the adult heart

Heart size is not significantly altered by either the gain or loss of α1G TTCCs, in spite of the fact that the α1GDT mice have a modest hypercontractile phenotype [18]. The fact that α1G−/− myocytes are smaller, and α1GDT myocytes are larger than WT suggests that TTCCs influence myocyte growth. This is clearly a complicated issue since in pathological stress α1GDT hearts have less hypertrophy while α1G−/− have more hypertrophy [27]. α1G−/− myocytes also had a significantly higher fraction of mononucleated myocytes. These data suggest that the number and nucleation pattern of myocytes within the adult heart are influenced by the absence or presence of TTCCs. We speculate that neonatal α1G−/− myocytes could retain a low level of proliferative activity resulting in the increased number of small myocytes which we found in the adult heart, while α1GDT myocytes might exit from the cell cycle at a faster than a normal rate, resulting in fewer, larger myocytes in the adult heart. These issues cannot be resolved with the studies performed here and deserve additional study. Importantly there is no evidence for cardiac fibrosis or any pathological phenotypes in any of the mouse models used in this study [27]. The presence of normal cardiac histology allows us to approximate the relative myocyte number in each of these three mouse groups, as we have done in previous studies [35]. Our findings suggest that α1G−/− hearts have the greatest number of myocytes (per unit mass) while α1GDT hearts have the smallest number of myocytes.

The major findings of this study are all consistent with the idea that Ca^2+^ influx through TTCCs regulates myocyte growth as well as the transition from mono- to bi-nucleation and the associated exit of myocytes from the cell cycle soon after birth. α1G−/− myocytes appear to have a reduced rate of cell cycle withdrawal, resulting in an adult heart with a normal size but with an increased number of small, mononucleated myocytes. α1GDT myocytes are larger than normal, yet their hearts are also normal in size. This is a complex phenotype that could result from a premature exit of myocytes from the cell cycle, resulting in a reduced number of myocytes per unit mass. The α1GDT phenotype is more complicated because these myocytes (and hearts) are hypercontractile [27] and this could induce secondary reactive phenotypic features.

There are a number of limitations in our study that largely result from the fact that it is challenging to study cell cycle activity in the heart and to determine the source of new cardiac myocytes. Our results are consistent with the idea that there may be a greater number of smaller, mononucleated myocytes in the α1G−/− adult heart. It is unclear if the basis for this is enhanced myocyte proliferation and if this occurs during fetal life or during the maturation of the adult heart. How α1G TTCCs are linked to the alterations in myocyte size, nucleation and the capacity for proliferative activity is, in our view, an issue deserving study.

In summary, these experiments suggest that TTCCs are expressed transiently in cardiac myocytes and the resultant Ca^2+^ influx is involved in the regulation of myocyte growth and the exit of neonatal myocytes from the cell cycle. Understanding the molecular bases of these processes could lead to novel strategies to coax myocytes into or out of the cell cycle to generate new cardiac myocytes in times of stress.

## Supplementary Material

1
