# Supplementary material for: T-type Ca2+ channels regulate the exit of cardiac myocytes from the cell cycle after birth
Source: J Mol Cell Cardiol. Author manuscript; Available in PMC 2014 Sep 1. (PMC3888788; doi:10.1016/j.yjmcc.2013.05.016)
Supplement: 1 [file NIHMS526067-supplement-1.pptx]

## Slide 1
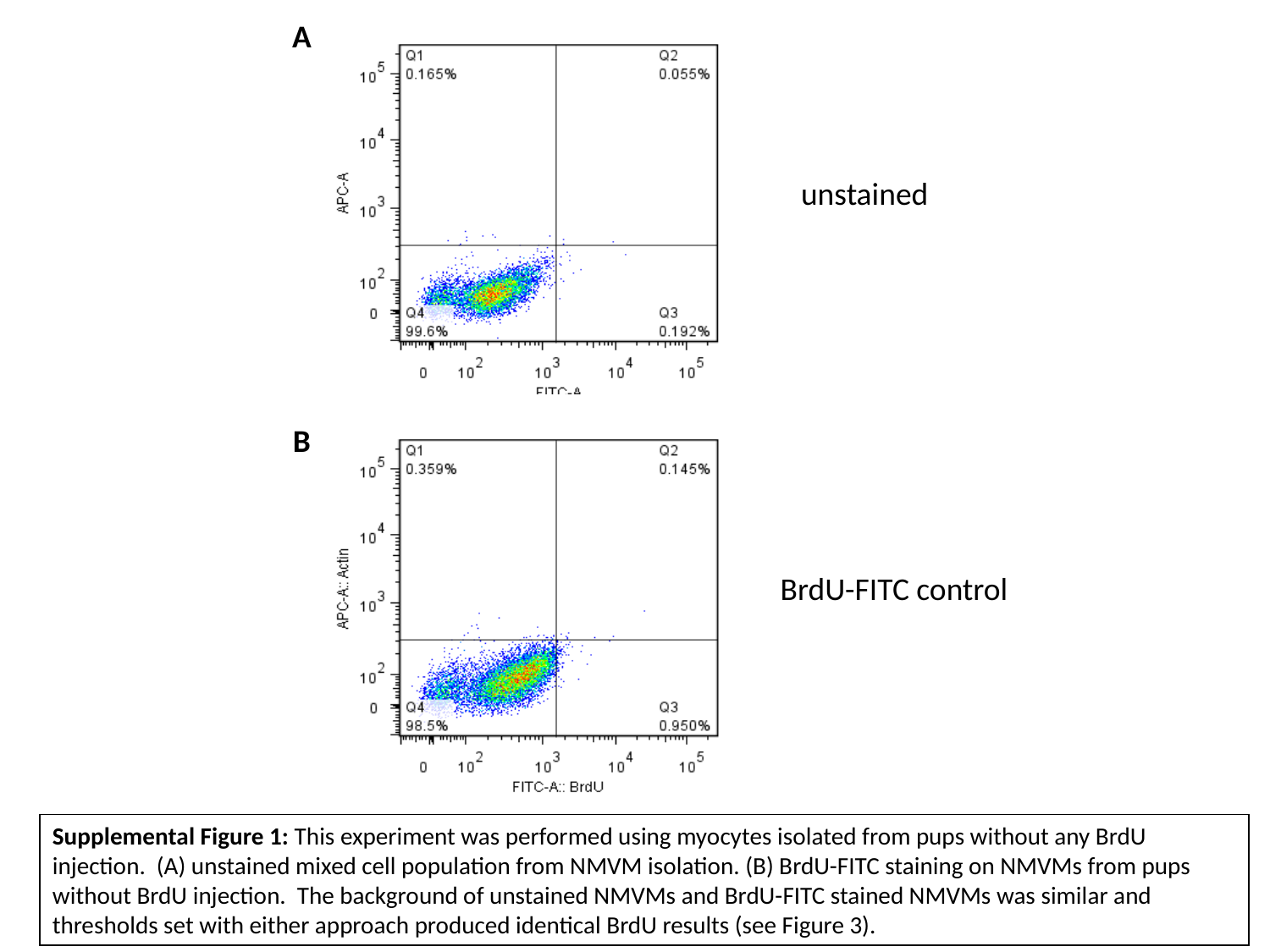

A
unstained
B
BrdU-FITC control
Supplemental Figure 1: This experiment was performed using myocytes isolated from pups without any BrdU injection. (A) unstained mixed cell population from NMVM isolation. (B) BrdU-FITC staining on NMVMs from pups without BrdU injection. The background of unstained NMVMs and BrdU-FITC stained NMVMs was similar and thresholds set with either approach produced identical BrdU results (see Figure 3).

## Slide 2
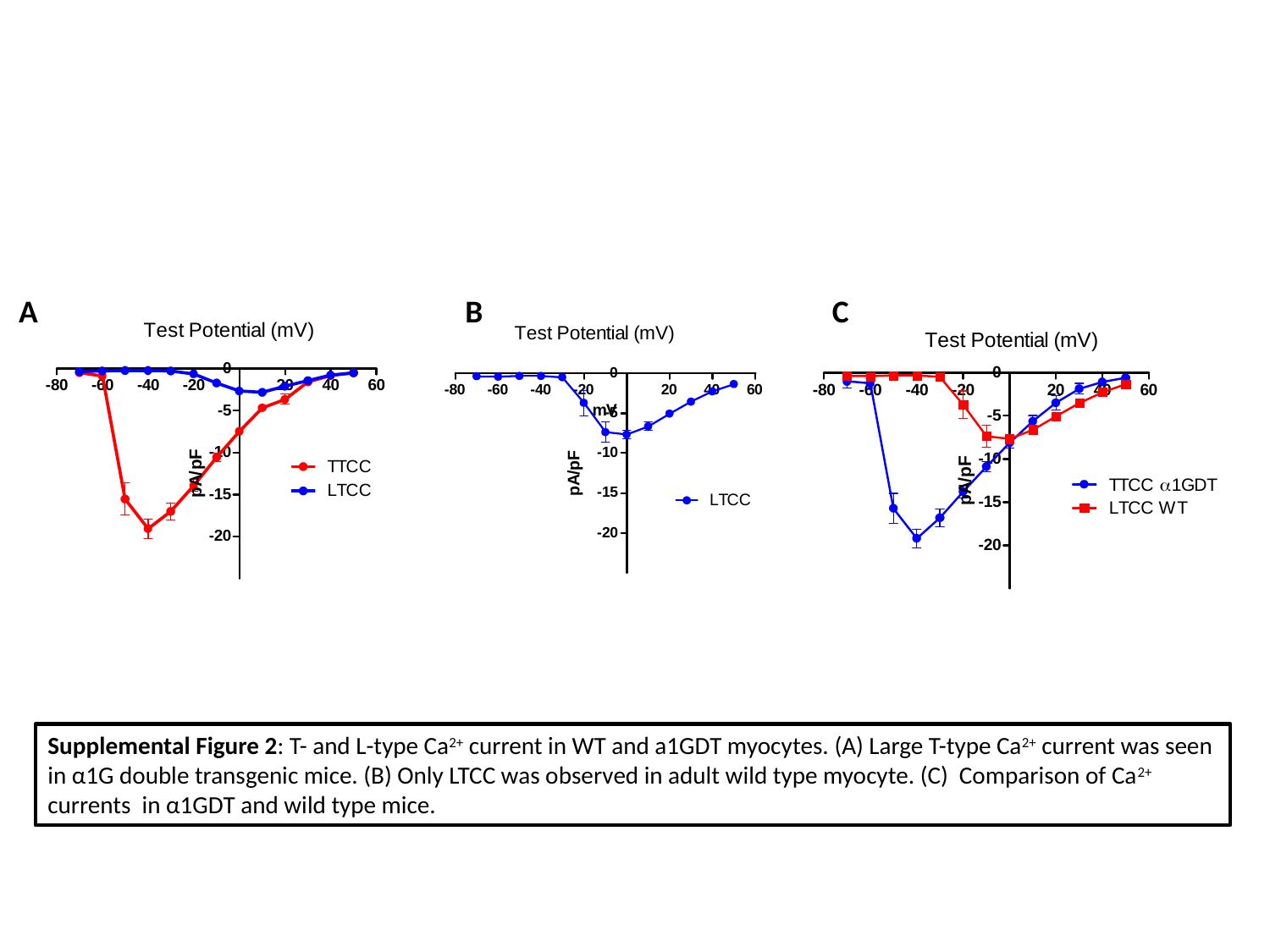

A
B
C
Supplemental Figure 2: T- and L-type Ca2+ current in WT and a1GDT myocytes. (A) Large T-type Ca2+ current was seen in α1G double transgenic mice. (B) Only LTCC was observed in adult wild type myocyte. (C) Comparison of Ca2+ currents in α1GDT and wild type mice.
